# The Timing and Effort of Lexical Access in Natural and Degraded Speech

**DOI:** 10.3389/fpsyg.2016.00398

**Published:** 2016-03-30

**Authors:** Anita E. Wagner, Paolo Toffanin, Deniz Başkent

**Affiliations:** ^1^Department of Otorhinolaryngology/Head and Neck Surgery, University Medical Center Groningen, University of GroningenGroningen, Netherlands; ^2^Graduate School of Medical Sciences, School of Behavioral and Cognitive Neuroscience, University of GroningenGroningen, Netherlands

**Keywords:** time-course of speech perception, speech perception in adverse communicative situations, cochlear implants, pupillometry, lexical processing

## Abstract

Understanding speech is effortless in ideal situations, and although adverse conditions, such as caused by hearing impairment, often render it an effortful task, they do not necessarily suspend speech comprehension. A prime example of this is speech perception by cochlear implant users, whose hearing prostheses transmit speech as a significantly degraded signal. It is yet unknown how mechanisms of speech processing deal with such degraded signals, and whether they are affected by effortful processing of speech. This paper compares the automatic process of lexical competition between natural and degraded speech, and combines gaze fixations, which capture the course of lexical disambiguation, with pupillometry, which quantifies the mental effort involved in processing speech. Listeners’ ocular responses were recorded during disambiguation of lexical embeddings with matching and mismatching durational cues. Durational cues were selected due to their substantial role in listeners’ quick limitation of the number of lexical candidates for lexical access in natural speech. Results showed that lexical competition increased mental effort in processing natural stimuli in particular in presence of mismatching cues. Signal degradation reduced listeners’ ability to quickly integrate durational cues in lexical selection, and delayed and prolonged lexical competition. The effort of processing degraded speech was increased overall, and because it had its sources at the pre-lexical level this effect can be attributed to listening to degraded speech rather than to lexical disambiguation. In sum, the course of lexical competition was largely comparable for natural and degraded speech, but showed crucial shifts in timing, and different sources of increased mental effort. We argue that well-timed progress of information from sensory to pre-lexical and lexical stages of processing, which is the result of perceptual adaptation during speech development, is the reason why in ideal situations speech is perceived as an undemanding task. Degradation of the signal or the receiver channel can quickly bring this well-adjusted timing out of balance and lead to increase in mental effort. Incomplete and effortful processing at the early pre-lexical stages has its consequences on lexical processing as it adds uncertainty to the forming and revising of lexical hypotheses.

## Introduction

Understanding speech involves the rapid translation of acoustic information into meaning. The time course in which listeners extract phonetic information and map it onto their mental representations has been extensively studied in ideal listening conditions (e.g., Allopenna et al., 1998; Dahan and Tanenhaus, 2004). It is, however, less well understood how degraded signals, such as speech transmitted via cochlear implants (CIs) – hearing prostheses that allow profoundly deaf listeners to regain access to speech perception – find their way into the mental lexicon. When noisy surroundings or hearing impairment complicate speech comprehension, effortful processing is the first noticeable consequence. This paper investigates how signal degradation affects the time course of lexical access, as reflected in listeners’ gaze fixations, and how lexical processing employs mental resources, as reflected in pupil dilation.

In ideal conditions, understanding speech is a prime example of an automatic perceptual process that takes its course without our attention. We can understand speech and at the same time engage in parallel activities. What enables this efficient processing is the seamless transfer of information within a hierarchy of pre-lexical and lexical decoding stages. Models of speech perception (e.g., TRACE: McClelland and Elman, 1986; Shortlist: Norris, 1994; Shortlist B: Norris and McQueen, 2008) describe pre-lexical and lexical processing as automatic. Evidence that listeners process speech even in absence of conscious awareness (Davis et al., 2007) further supports this notion. Unlike speech perception in ideal situations, the processing of degraded speech draws more strongly on attentional resources (Wild et al., 2012), and can lead to mental fatigue (Hornsby, 2013).

Increased effort during speech perception, sometimes also referred to as mental fatigue (for a distinction of these terms see McGarrigle et al., 2014), is often reported by users of CIs (Noble et al., 2008). Compared to natural speech the signal transmitted via CIs is strongly degraded in its spectrotemporal form. Following implantation listeners need to adapt their processing of speech to this specific transmission, and despite reaching relatively successful speech understanding on average, many listeners describe speech perception to be more tiresome. The symptom of greater effort during speech perception has also been reported for hearing impaired listeners (Kramer et al., 1997), and hearing-aid users (Hornsby, 2013).

Audiological assessment methods are traditionally based on measures of intelligibility and no standard tests exist for quantifying effort. Mental effort is first and foremost listeners’ impression, but it may affect automatic mechanisms underlying speech perception, and bottlenecks within these mechanisms can increase effort even further. Recently, there has been an increase in interest in pupillometry as an objective measure of mental effort in speech perception (Kramer et al., 1997; Zekveld et al., 2014; Koelewijn et al., 2015). Pupillometry has confirmed itself as a method to study the subconscious use of attentional resources in cognition since Hess and Polt (1960), and Kahneman and Beatty (1966). These classical studies established that the dilation of the human pupil does not only reflect adaptation to changes in luminance, in the timescale of 200–500 ms (Ellis, 1981), but also a slower evolving response to mental effort, in the timescale of above 900 ms (Hoeks and Levelt, 1993). Since then, pupillometry has been applied to study cognitive processes, such as those related to memory load (e.g., Hess and Polt, 1960) or attention (Hoeks and Levelt, 1993). Whereas it is accepted that increased pupil dilation reflects increased processing, the sources of pupil dilation can be attributed to mental effort (Hess and Polt, 1964), controlled attention (Hoeks and Levelt, 1993; Koelewijn et al., 2015), automatic attention (Libby et al., 1973), or engagement in a task (Kahneman and Beatty, 1966; Kang et al., 2014). There is no clear-cut distinction between effort and attention, and some models of cognitive resources see a close correlation between effortful processing and increased demands on attention (Kahneman, 1973). For speech perception, pupillometry has been applied to study listening effort under divided attention (Koelewijn et al., 2015), listening effort (Zekveld et al., 2014; Winn et al., 2015), and speech perception training (Kuchinsky et al., 2014). Greater pupil dilation has been found to reflect both auditory and cognitive aspects of processing speech in challenging conditions (Zekveld et al., 2014).

Effortless processing of speech in optimal conditions is based on experience with the signal, and on the consequential fine attunement of the perceptual system to the regular and common patterns in the listener’s native language (Cutler, 2012). Language-specific processing of speech starts with early and subconscious perceptual organization of acoustic cues (e.g., Kuhl, 1991; Iverson et al., 2003), and continues with semantic and pragmatic integration of meaning into the context of a conversation (Kamide et al., 2003). This fine adjustment takes place during speech development (Kuhl, 1991; Best, 1995), and ensures the almost instant processing of the speech signal, as speech needs to be processed in real time. A delay in the processing of speech on pre-lexical and lexical stages will decrease the automaticity of speech perception and may increase mental effort. Gaskell and colleagues (Cleland et al., 2006, 2012) show the importance of well-timed lexical processing in a series of experiments deploying the Psychological Refractory Period. Early stages of speech processing, such as integration of cues to phonemic identification appear to take place without drawing upon central resources (Gaskell et al., 2008). Accessing the meaning of words, however, has been found to create a bottleneck, which sets a limit on the processing of subsequently presented tasks (Cleland et al., 2012). Degradation of the signal may affect the fine timing of processing even further.

The aim of the present study is to track the timing of lexical access in natural and degraded speech, and to study whether and how this processing interacts with mental effort. We hypothesize that degradation will affect the automaticity of processing speech and delay the timing of processing information at pre-lexical and lexical levels. The time course of lexical access has been studied by means of eye-tracking (e.g., Allopenna et al., 1998; Dahan et al., 2002). This paradigm is based on the over the decades replicated finding that listeners’ gaze fixations to pictures displayed on a screen are driven by auditory speech stimuli: listeners spontaneously fixate the object that is being referred to in the signal they hear (Cooper, 1974). This paradigm thus captures the time course of lexical decision-making. Previous eye-tracking studies have documented listener’s fast integration of detailed phonetic and semantic information and how this information modulates their lexical decisions (Dahan et al., 2002; Salverda et al., 2003; Dahan and Gaskell, 2007).

The process of interest in this paper is lexical competition, which is the short-lived interval during which the heard signal matches multiple lexical entries, and the perceptual system allows multiple lexical candidates to compete for the best match to the signal. Listeners, not knowing the intended word beforehand, subconsciously and for splits of milliseconds consider multiple words that have overlapping phonological forms. This includes homonyms (e.g., pair and pear), lexical embeddings (e.g., paint in *paint*ing), and words that can occur across word boundaries (e.g., can in bla*ck an*d blue). Models of speech perception see lexical competition as integral part of lexical access (for a recent discussion on this debate see McMurray et al., 2009).

The present experiment adapts the design by Salverda et al. (2003), who studied the time course of disambiguation of words embedded in other words (e.g., pan in panda) in Dutch. These authors found that listeners’ gaze fixations during the processing of lexical embeddings are guided by the durational differences between syllables in monosyllabic versus polysyllabic words. The lengthening of syllables in boundary position makes the monosyllabic word, e.g., *pan*, longer than the phonologically overlapping syllable in the polysyllabic word *pan*da. To study the effect of the durational cues on lexical decision, Salverda et al. (2003) manipulated the duration of the first syllable by cross-splicing monosyllabic words into polysyllabic targets. This manipulation will be part of our experiment, as well as the second manipulation of signal degradation that simulates the signals transmitted via CIs. We will record the time-course of lexical disambiguation in natural and degraded stimuli. The durational manipulation is crucial in combination with the specific degradation applied because while CIs strongly degrade the signal in its spectrotemporal details they reliably transmit the durational relations in speech (Vavatzanidis et al., 2015). This means that listeners can pick up on the durational cues for both degraded and natural speech stimuli. In order to also get insight into the mental effort involved in lexical access we will record listeners’ pupil dilation alongside the fixations.

Pupil dilation will give us insight into the mental effort involved in the processing of degraded versus natural speech. The measure of mental effort captured in pupil dilation combined with gaze fixations can reflect processing bottlenecks, or the accumulated effort resulting from ill-adjusted timing between processing stages. However, pupil dilation may also indicate the engagement in a task, or the recruitment of attentional resources. The manifold sources of pupil dilation have led to some ambiguity in the use of terms. In this paper we will use the term ‘mental effort’ to describe our results. However, we are aware that automatic attentional allocation can play a role in the regulation of cognitive processes (Posner, 1992), as is the perception of speech. Furthermore, the capacity model by Kahneman (1973) sees a close connection between attention and mental effort. In this model, tasks compete for processing resources with automatic tasks requiring no attention and little effort. Although speech perception is often described as automatic, this automaticity is granted mainly when listening to native speech. Listening to a foreign but familiar language already demands more attention and effort in processing. There is growing evidence for the involvement of attentional resources, in particular when processing speech in adverse conditions (Mattys et al., 2009; Wild et al., 2012). Even for the perception of natural signals attention has been found to not only facilitate segregation of speakers (Kerlin et al., 2010), but also to share resources with parallel tasks, such as performing memory-related tests while suppressing irrelevant non-speech sounds (Sörqvist et al., 2012).

Three questions stand in focus of the present study. (1) Does the time course of lexical disambiguation, as captured by gaze fixations, differ between the processing of natural versus degraded speech? (2) Does lexical competition involve an increase in mental effort, as captured in listeners’ pupil dilation? (3) Does processing of degraded speech show a comparable course of changes in mental effort to natural speech? Based on our working hypothesis that timing between the processing stages is crucial for automatic and effortless perception we assume that there will be differences in the time course of processing natural versus degraded speech. A hint into a similar direction has been reported by Farris-Trimble et al. (2014). Regarding question two, we expect to find a difference in pupil dilation for the processing of degraded versus natural speech. We do not expect lexical competition in natural speech to employ mental resources, since speech perception is an automatic process. Our experimental stimuli, however, contain misleading cues that will force listeners to revise their lexical hypotheses, and we expect that mental resources may then be recruited. Coming to our third question, we expect to observe more effort in processing degraded speech, as it has previously been reported (Zekveld et al., 2014; Winn et al., 2015). However, it is still an open question whether processing degraded speech *per se* already depletes mental resources allocated to speech perception or whether an additive effect of lexical competition can also be observed. Should the course of fixation between degraded and natural speech indeed differ, as we hypothesize, then the recruitment of mental resources or the course of effort visible in pupil dilation might differ as well.

## Experiment

### Method

#### Participants

Seventy-three normal hearing volunteers, aged between 20 and 31 years (mean age 24), participated in this study. None of them reported any known hearing or learning difficulties, and they all had normal or corrected-to-normal vision. Their hearing thresholds were normal, i.e., below 20 dB HL at the audiometric frequencies between 500 and 8000 kHz. Half of the volunteers were randomly assigned to participate in the task with natural speech (NS), and the other half with degraded speech (DS). Before the experiment started, the participants signed a written consent form for the study as approved by the Medical Ethical Committee of the University Medical Centre Groningen. The volunteers received either course credits or a small honorarium for their participation.

#### Stimuli

The materials consisted of 26 critical items, which were borrowed from Salverda et al. (2003). These were polysyllabic Dutch words, which were paired with initially embedded, thus phonologically overlapping, monosyllabic words as competitors. The stimuli set contained next to the critical items also 40 filler items, partly again borrowed from Salverda et al. (2003) and partly constructed for this study. The fillers were selected based on two criteria: their syllabic structure, and presence of embedded words. Seven of the fillers were polysyllabic and 33 were monosyllabic words, thus allowing us to balance the distribution of short and longer words as targets throughout the experiment. Twenty of the fillers did not contain a competitor, ten of the fillers were monosyllabic words that were paired with polysyllabic words in which they were embedded in initial position. The remaining ten filler targets were monosyllabic words paired with polysyllabic words that embedded them in final position.

For all the materials, the sentence context was neutral and revealed no semantic information about the target. A female native speaker of Dutch with no prominent regional accent recorded the sentences in blocks of paired sentences. The speaker was instructed to pronounce the sentences clearly but in a natural manner. For each pair of target- and competitor items three sentences were recorded. The sentence containing the polysyllabic, thus embedding, target (e.g., bokser [boxer]) was recorded twice. Only one instance of the sentence with the monosyllabic, hence embedded, competitor (e.g., bok [goat] is embedded in bokser) was necessary to construct the materials. The initial part of both sentences was identical, and the monosyllabic (competitor) word was always followed by words that matched the phonological, prosodical, and stress pattern of the target sentence as closely as possible. For instance, for the target word ‘bokser’ the sentence *Wij wisten wel dat de oude bokser gestopt was* [We all knew that the old boxer retired] was paired with the sentence *Wij wisten wel dat de oude bok suffig was* [We all knew that the old goat was drowsy]. In order to accentuate the durational differences that were driving listeners’ gaze fixations in the study by Salverda et al. (2003), words following the monosyllabic words were stressed on their first syllable. Due to final lengthening words preceding a stressed position are produced as longer. This allows us to ascertain that the durational cues were audible in the degraded signals. The differences in length between the embedded syllables and the syllables in the polysyllabic words ranged between 20 and 120 ms, with a mean of 65 ms.

All materials were subjected to a splicing procedure, in analogy to Salverda et al. (2003). An example of the procedure is shown in **Table 1**. The acoustic manipulation was implemented in PRAAT (Boersma and Weenink, 2013), and consisted of combining the three sentences recorded per critical pair in order to create two experimental conditions. The sentences were divided into two parts: the initial part contained the sentence up to either the first syllable of the polysyllabic word, or the end of the monosyllabic word; the second part contained the second syllable of the polysyllabic word until the end of the sentence. In Condition 1 (target-matching cues) the first part of the polysyllabic sentence was combined with the second part of the second recording of the same polysyllabic sentence. In Condition 2 (target-mismatching cues) the first part of the monosyllabic sentence was combined with the same second part of the polysyllabic sentence as in Condition 1. This resulted in Condition 1 having the durational pattern typical for the polysyllabic word, and Condition 2 having the durational pattern typical for the monosyllabic word, where this pattern, however, was then violated when the second syllable extracted from the target word was presented.

**Table 1:** An example of the recorded sentences, and the splicing manipulation applied to create the target-matching and target-mismatching condition.

**Recorded materials**	Sentence 1	We wisten wel dat die oude BOKSER gestopt was
	Sentence 2	We wisten wel dat die oude BOKSER gestopt was
	Sentence 3	We wisten wel dat die oude BOK suffig was
**Condition 1**	Target-matching duration	We wisten wel dat die oude BOK⋅SER gestopt was
**Condition 2**	Target-matching duration	We wisten wel dat die oude BOK⋅SER gestopt was


**FIGURE 1:**
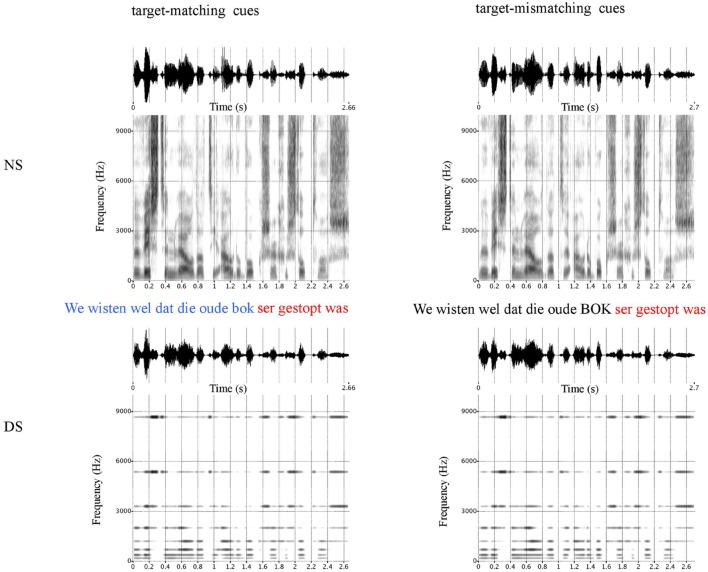
**Examples of the stimuli for the two experimental conditions for natural speech (NS) and degraded speech (DS).** Depicted are the waveforms and spectrograms of the natural stimuli **(top)**, and the degraded stimuli **(bottom)**, with target-matching **(left)** and target-mismatching duration cues **(right)**.

The degradation in the form of acoustic CI simulation was performed by sinusoid vocoding the speech signal with eight channels, and implemented in MATLAB. The decision to create vocoded stimuli with eight channels is based on the finding that increasing the number of channels improves speech perception of CI users up to seven channels and then plateaus (Friesen et al., 2001). The stimulus signal within the frequency range of 100 Hz – 10 kHz was bandpass filtered into eight frequency bands. The intervals between these eight channels were chosen to be equally spaced based with regards to the basilar membrane using Greenwood’s mapping function (Greenwood, 1990). The amplitude envelopes of these channels were extracted in each frequency band, by first half-wave rectification, then low-pass filtering (4th order Butterworth) the band-limited signal at 300 Hz. The simulated speech was obtained by summing up sinusoids at a frequency matching the center frequency of each band modulated with the extracted envelopes. **Figure 1** displays the spectrograms of an experimental sentence in its natural (NS) and vocoded form (DS), for the stimuli with target-matching (left panel) and competitor-matching durational cues (right panel).

#### Apparatus and Presentation

The eye-tracker SIM Eyelink 500, with a sampling rate of 250 Hz was used. This head mounted eye-tracker contains two small cameras, which can be aligned with the participants’ pupil to track the pupil’s movements as well as its size continuously during the experiment. The listeners were seated in front of a 19-inch monitor, within a distance of about 50–60 cm from the screen. The stimuli were presented via a speaker in sound attenuated room at a comfortable level of about 65 dB SPL. The lighting in this room was kept constant throughout the experiment.

**FIGURE 2:**
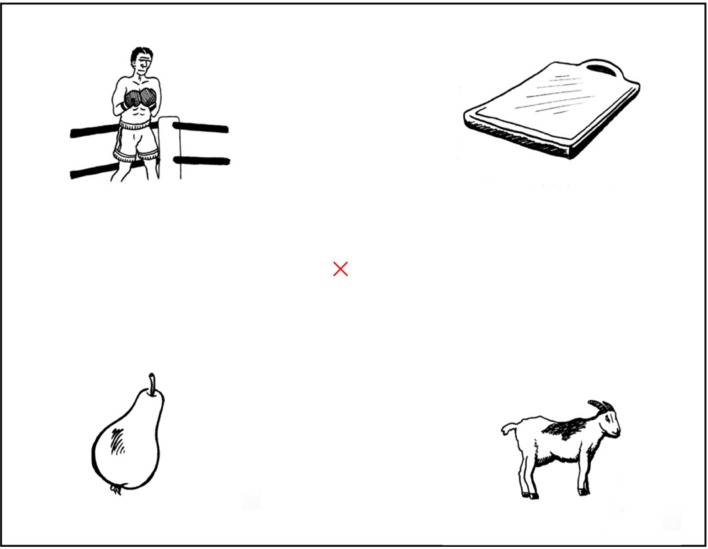
**Example of the display presented to the participants, with bok (goat) as competitor for the target bokser (boxer)**.

For the display, black and white line drawings were made for the purpose of this study, and validated through consistent naming by Dutch native speakers. For the presentation of the pictures a virtual grid was created to divide the screen into three horizontal and three vertical bars. A red cross appeared centered in the middle quadrant resulting from the 3^∗^3 partition of the screen, and the four pictures were centered in the four external quadrants on the grid. An example of a display with *bokser* (boxer) as target and *bok* (goat) as competitor are shown in **Figure 2**. The pictures of the 26 critical items were always presented with the respective monosyllabic competitor and two phonologically and semantically unrelated distractors (see Supplementary Material). Twenty filler items were presented with 3 unrelated distractors, 10 monosyllabic fillers were presented with their word-final embedding (target: *bel;* competitor: li*belle*), and 10 with the monosyllabic embedding competitor (e.g., target: *mand*; competitor: *mand*arijn).

#### Procedure

Before the experiment all participants were familiarized with all the pictures to ensure that they identified them as intended. The pictures were presented to the participants who named them, and were then told the intended name in case of a mismatch between the word used in the experiment and their identification (for instance to clarify synonyms, such as couch and sofa). Participants assigned to the DS condition were familiarized with the sort of degradation used in the experiment. They were presented with at least 30 degraded sentences and were asked to click on the correct sentence that was written amongst 10 sentences on the screen. During this phase participants were allowed to listen to these sentences as often as they wanted. After that the eye-tracker was mounted and calibrated.

Before the data collection started, participants performed four practice trials during which the participant could always refer to the experimenter to ask for instructions. Each trial consisted of a red cross appearing on the screen for 500 ms, followed by the visual display of the four pictures, and simultaneous auditory presentation of the sentence. Participants were instructed to listen to the stimuli and to click on the object mentioned in the sentence. They were also instructed to blink only between the trials, while the word “Blink” appeared on the screen. After each of the blinking pauses participants could progress on a self–paced basis. After every five trials a recalibration screen appeared, to make sure the eye-tracker did not lose track of the pupil. The experiment lasted on average 15–20 min, and consisted of 62 trials, 26 of which were critical trials. The session needed to realize the experimental protocol, including initial information of the participant, the hearing screening, familiarization with the pictures and the degradation, and debriefing lasted about 1 h.

#### Data Analysis

Listeners correctly clicked on the target in 95% of the trials. Trials in which participants failed to identify the intended target word or with blinks longer than 300 ms were excluded from the analysis (on average two trials per participant). The SR Eyelink 500 records blinks as data points with x–y coordinates and pupil size information. Blinks shorter than 300 ms were linearly interpolated based on the median of 25 samples recorded before and after the blink.

The data of two participants were excluded from the analysis because their number of misidentification of the target together with trials containing blinks longer than 300 ms summed up to 50% of the trials. In addition, the data of four other participants were discarded due to computer or calibration failures. Following this, the data set contained the recordings of 67 participants, 35 of which took part in DS and 32 in NS.

The statistical analysis of the data is based on the interval between 200 and 2000 ms after word onset. The first 200 ms after the onset of the target are needed to plan and perform the eye movement triggered by an auditory stimulus for a display with multiple pictures (Hallett, 1986), and participants always clicked on the target within the interval of 2 s. The statistical analysis of the gaze fixation will focus on the fixations toward the competitor, since these time curves give insight into how quickly listeners use duration as cue, and how it modulates their lexical decision.

Pupil size data were recorded as pupil area alongside fixations at each sample point. However, eye movements may affect the measurement of pupil size. To ensure that such measurement artifacts do not introduce differences between the experimental conditions, we counted the number of fixations per trial. Within our analysis window of 200–2000 ms we counted on average three fixations. We found no differences between the experimental conditions, neither between filler items nor critical items. Thus if eye movements affected the measurements of pupil size, they did so equally for all conditions. Our approach of combining gaze fixation data with pupillary responses is similar to Klingner (2010), and following his report we also visually inspected the course of pupil dilation across movements for drastic changes in pupil size that would signal measurement errors due to movements. Within our analysis window we did not see such jumps in pupil size. To calculate pupil size changes related to the presentation of stimuli – Event Related Pupil Dilation (ERPD) – we time-locked the pupil size data to the presentation of the target word, corrected it to a baseline immediately preceding the target word, and then normalized the values to correct for individual differences in pupil size, according to the following Equation.

$$\%ERPD=\frac{observation-baseline}{baseline}*100$$

To address the questions of whether lexical competition leads to increased pupil dilation and whether the course of pupil dilation is comparable for degraded and NS, we used two different baselines to compute two percentage changes in ERPD. Baseline 1 will enable us to study the pupil size within the time window of lexical competition. To specifically observe the effect of our experimental manipulation, and to limit other sources that can lead to changes in pupil dilation, baseline 1 is the interval that immediately precedes the manipulation. Baseline 2 will examine whether potential effects of lexical competition on pupil dilation are comparable across groups. More effortful processing of DS (Zekveld et al., 2014; Winn et al., 2015) implies an increase in pupil size due to the higher demands when processing DS. Baseline 2 must thus be free of any differences in pupil size between groups of participants assigned either to DS or NS. Therefore, baseline 2 is the average pupil size in the interval preceding the very first sentence in the experiment, where the average pupil size was not significantly different between the groups. Specifically, baseline 1 is the average pupil size within the interval of 200-ms preceding the target word within the sentence, and is computed separately per participant and trial. This value was then inserted into the above equation. Whereas baseline 1 focuses on the processing of lexical competition, the individual normalization per trial may potentially conceal group difference (DS versus NS) in the baseline itself. Baseline 2 is the average pupil size in the interval of 200 ms at the very beginning of the experiment. This value was then inserted into the above equation. Percentage change in ERDP computed from baseline 2 encompasses all the cognitive processes that take place while solving the experimental task – processing speech–, and provides a reliable baseline for the effort induced by the experiment.

### Statistical Analyses

#### Fixations

The probability of listeners fixating the competitor was analyzed by means of logistic growth curves analysis models (Mirman, 2014). R (R Core Team, 2013) with lme4 package (Bates et al., 2014) was used to model the time curves of fixations as fourth order polynomials within the time window of 200–1800 ms after target word onset. The time course curves were described in four terms: intercept, the overall slope of the curve, the width of the rise and fall around the inflection, and the steepness of the curvature at the tails. The probability of fixations along the time course was modeled as a function of Presentation (NS versus DS), Condition (target-matching duration versus target-mismatching duration) and the possible interactions between these two factors and all four terms describing the curves. As random effect we included individual variation among participants on all four terms describing the time curve. Model comparison was used to estimate the contribution of individual predictors to the fit of the model. For this, individual fixed effects were sequentially added, and the change in the model fit was evaluated by means of likelihood ratio test.

#### Pupil dilation

The pupil size data, as captured by the ERPD, was also analyzed by means of Growth Curve Analysis, as time curves of pupil dilation. The courses of dilation were analyzed as polynomial curves of third order, since the fourth order turned out to be redundant to the description of the curve functions. The terms describing the curves are: intercept, the slope of the function, and a coefficient for the curvature around the inflection point. The statistical models included the terms describing the curves, an interaction of these three terms with the experimental conditions (target-matching versus target-mismatching cues) and presentation condition (NS versus DS). To account for individual variation also random effects of the terms describing the curve were included per participant.

### Results

#### Fixations

**FIGURE 3:**
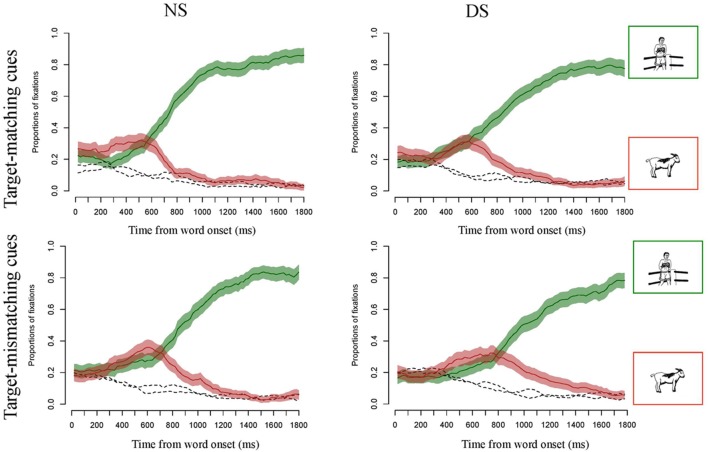
**Curves of proportions of gaze fixation over time for the target-matching and target-mismatching conditions, when presented with natural (NS) and degraded speech (DS).** The green lines show the proportion of fixations averaged across participants and items and the 95% confidence intervals for target fixations, red lines show the same for competitor fixations, and the dashed black lines show fixations to the distractors.

**Figure 3** displays the time-curves of fixations to all four pictures for both target-matching (top panels) and target-mismatching (bottom panels) conditions and split by groups presented with NS (left panels) and DS (right panels). This figure shows proportions of fixations averaged across participants, and the 95% confidence intervals for the fixations to the target and competitor. A comparison between the top and the bottom panels gives insight into how the mismatching duration led listeners’ gaze fixations to the competitor. The point at which the fixations curves to competitor cross with the curve for the target signals the point at which on average the target won the process of lexical competition. In the presentation with NS the difference in time between the two conditions is about 120 ms. This is the maximum duration needed for the disambiguating acoustic information (i.e., the second syllable of the target word) to come in. A comparison between NS and DS shows that this point of disambiguation was delayed for DS on average for some 40 ms for the target-matching stimuli, and of 140 ms in the target-mismatching condition. For the presentation with DS, we find a greater difference in timing of lexical disambiguation due to acoustic information: here, the difference between the target matching (right upper panel) and mismatching cues (right bottom panel) is above 200 ms.

**FIGURE 4:**
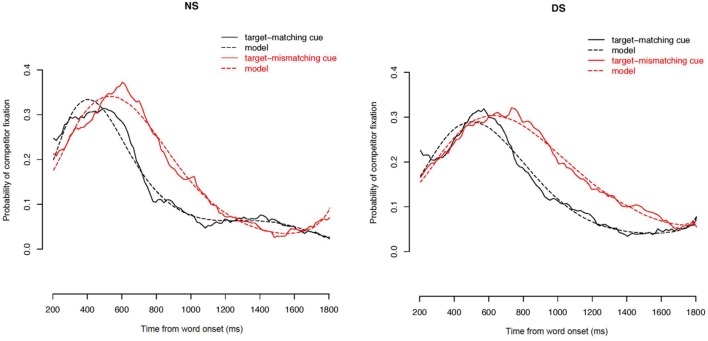
**Effect of mismatching cues in NS **(left)** and DS **(right)**.** The probability of fixations toward the competitor in target matching (black), and target mismatching stimuli (red) is displayed for NS and DS conditions, in the averaged data (solid lines), and in the data fitted by the statistical model (dashed).

Of particular interest for this study is the question of statistical significance of the interactions between condition and experiment and the terms describing the course of the curves. These interactions were significant (see **Table 2** for a summary of the model estimates). **Figure 4** displays the effects of condition on the time curves of fixations toward the competitor for NS and DS, respectively. These figures display the probability of fixations toward the competitor on the averaged data (solid lines), and on the data as fitted by the statistical model (dashed lines). The interaction between the intercept of the curve and Condition and Presentation [χ^2^(3) = 185.28, *p* < 0.001] reflects that the difference between the areas underneath the curves for condition with target-matching versus target-mismatching cues was greater in the experiment with NS than with DS. This indicates that DS modified listeners’ ability to quickly integrate durational differences while forming their lexical hypotheses, and listeners’ gazes were slower directed toward the picture that best matched the acoustic information in the signal. This is also indicated by the three-way interaction with the slope of the curve [χ^2^(3) = 318.99, *p* < 0.001]: the time curves of fixations showed a steeper increase in the target-mismatching condition in NS than in DS, showing a faster reaction of listeners’ gazes to the durational cues. The three way interaction between the term describing the rise and fall of the curve around the central inflection [χ^2^(3) = 676.85, *p* < 0.001] describes the fact that the curve of fixations in the target-mismatching condition rose and fell significantly faster in NS than in DS. The three way interaction with the cubic term [χ^2^(3) = 471.77, *p* < 0.001] reflect the difference in the decline of fixations to competitor between the matching and mismatching condition in NS versus DS. This decline was slower for mismatching cues in DS.

**Table 2:** Summary of the estimates of the statistical model used for the analysis of gaze fixations to the competitor.

Factor	Estimate	Standard error	Significance
Curve Intercept ^∗^ condition ^∗^ experiment	11.76	1.13	<0.001
Curve slope ^∗^ condition ^∗^ experiment	20.63	1.18	<0.001
Curve rise and fall ^∗^ condition ^∗^ experiment	22.81	0.96	<0.001
Curve decline in tails ^∗^ condition ^∗^ experiment	9.26	0.60	<0.001


*Model:*

*Log-odds of fixations to competitor ∼ (Curve: intercept) ^∗^ (curve: slope) ^∗^ (curve: fall and rise around inflection peak) ^∗^ (curve: decline in tails) ^∗^ exp ^∗^ condition + random effects (all curve parameters per participant).*

*DS and target-matching condition are at the intercept.*

In sum, for the presentation with NS listeners’ gazes are quickly governed by the acoustic information in the signal: they fixate the competitor picture more often for stimuli that contain cues appropriate for the competitor. **Figure 4** (left panel) also shows a delay in the peak location of the fixation curves for competitor between the two conditions of about 120 ms in NS. Part of this delay is explained by the fact that the stimuli in the target-mismatching condition were longer by about 65 ms on average. This figure also shows that fixations to the competitor drop rapidly after the acoustic information that clearly disambiguates the target from competitor comes in. Hence, listeners very rapidly revise their initial lexical hypothesis that was based on the cues in the speech signal.

The right panel in **Figure 4** displays the differences in DS between the two conditions. In comparison with NS, the peak of fixations to competitor in the target-mismatching condition is not higher than the peak of fixations for the target matching items. Furthermore, the peak location for the target-mismatching condition is delayed even further, to more than 200 ms. This implies that listeners presented with DS did not show such a high sensitivity to durational cues as listeners presented with NS. Also, the integration of durational cues for lexical decision took longer, since the difference of 200 ms cannot be explained by the durational differences in the stimuli alone. The figure also visualizes the significant interactions with the third and fourth term of the time curve: the rise and fall of the competitor fixations curve is slower for DS than for NS, making for a shallower curve, and indicating that listeners decision on the lexical target was not as quick and not as certain in DS as in NS. The effect of uncertainty is further captured by the slower decline of competitor fixations at the tail of the curve: Even following the presentation of the clearly disambiguating second syllable of the word listeners still fixated the competitor to some degree.

#### Pupil Dilation

##### Baseline 1

**FIGURE 5:**
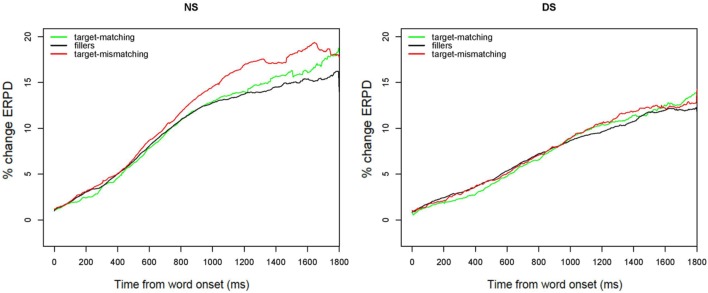
**Pupil dilation data time curves shown for NS **(left)** and DS **(right)** for target matching (green), target-mismatching (red) and filler stimuli (black)**.

The time-curves of the ERPD for the target-matching, target-mismatching and filler items are displayed in **Figure 5**. Note that the filler items did not elicit lexical competition. A visual comparison between the left panel (NS) and the right panel (DS) shows at first glance that the difference in pupil dilation between filler items and items inducing lexical competition was greater for NS than for DS. In analogy to the gaze fixations analysis, model comparison was used to estimate the significant contribution of the factors and interactions. The final model compared the dilation time curves across participant groups and conditions. The estimates of the final model are listed in **Table 3**. The three-way interaction between all the terms describing the curve, the presentation modes (NS versus DS), and the condition (fillers versus target-matching cues versus target-mismatching cues) was significant. The interaction between the first term – the intercept of the curve – [χ^2^(5) = 2051.6, *p* < 0.001] captures the differences in the areas underneath the curves across the conditions between NS and DS. The interaction with the second term – the slope – [χ^2^(5) = 86.37, *p* < 0.001] reflects the difference in the course of increase of pupil size between the conditions across NS and DS. The three-way interaction between the third term – the curvature around the peak – [χ^2^(5) = 173.48, *p* < 0.001] captures the release from increase in pupil dilation.

**Table 3:** Summary of the estimates of the statistical model used for the analysis of ERPD.

Factor	Estimate	Standard error	Significance
Curve Intercept ^∗^ condition(fillers versus matching cues) ^∗^ presentation (reference: NS)	-4.41	0.82	<0.001
Curve slope ^∗^ condition(fillers versus matching cues) ^∗^ presentation (reference: NS)	-4.90	0.82	<0.001
Curve rise and fall ^∗^ condition (fillers versus matching cues) ^∗^ presentation (reference: NS)	-1.87	0.82	<0.03
Curve Intercept ^∗^ condition(fillers versus mismatching cues)^∗^presentation (reference: NS)	14.41	0.82	<0.002
Curve slope ^∗^ condition(fillers versus mismatching cues) ^∗^ presentation (reference: NS)	-2.56	0.82	< 0.001
Curve rise and fall ^∗^condition(fillers versus mismatching cues)^∗^ presentation (reference: NS)	-3.11	0.82	<0.001
Curve Intercept^∗^condition (mismatching versus matching cues)^∗^presentation (reference: NS)	14.11	0.86	<0.001
Curve slope ^∗^ condition(mismatching versus matching cues) ^∗^ presentation (reference: NS)	-2.81	0.86	<0.002
Curve rise and fall ^∗^ condition(mismatching versus matching cues) ^∗^ presentation (reference: NS)	-3.22	0.86	<0.001


*Model: ERPD ∼ (Curve: intercept) ^∗^ (curve: slope) ^∗^ (curve: fall and rise around inflection peak) ^∗^ presentation (natural versus degraded) ^∗^ condition (target-matching versus target-mismatching versus filler items) + random effects (all curve parameters per participant).*

For NS (**Figure 5**, left panel), the ERPD curves show an increase over time as a function of lexical competition. The statistical analysis revealed that the target-matching curves differed from the target-mismatching curves on all terms describing the curves [χ^2^ (1) = 35.89, *p* < 0.001]. The curves for both conditions also differed significantly from the filler items in terms of slope [χ^2^(1) = 5.99, *p* < 0.001], curvature [χ^2^(1) = 8.65, *p* < 0.001], and area under the curve [χ^2^(1) = 16.53, *p* < 0.001]. This implies that pupil dilation captured the effect of lexical competition, and that dilation was significantly larger when the cues were mismatching the target. The right panel of **Figure 5** displays the pupil dilation time course for the stimuli with DS. These curves differed from each other only in terms of their intercept [χ^2^(1) = 18.3, *p* < 0.001], and both differed from the filler items only in the curvature of the function [χ^2^(1) = 5.84, *p* < 0.001]. This suggests that pupil dilation here did not capture effects of cue manipulation, and that the effect of lexical competition was only marginal.

**FIGURE 6:**
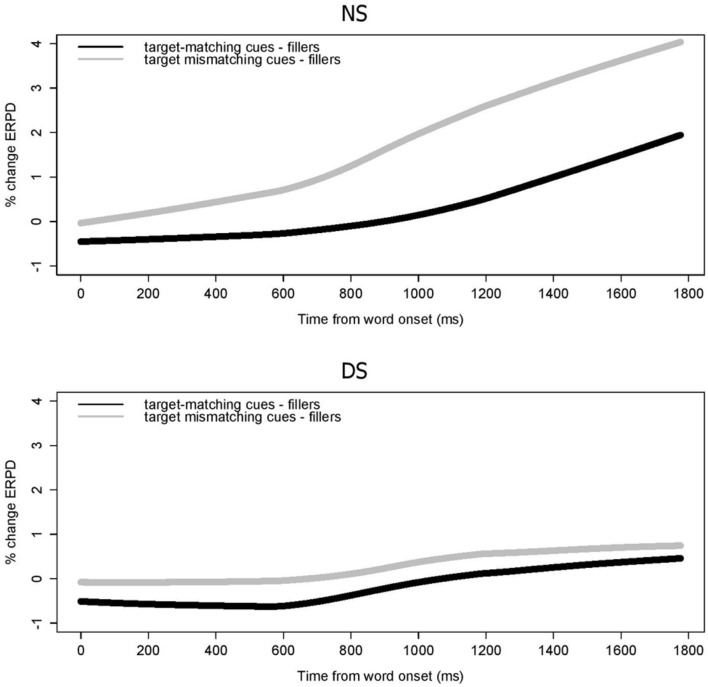
**Effect of lexical competition on mental effort in NS **(top)** and DS **(bottom)**, as captured by the difference in ERDP for critical experimental items and fillers.** The functions displayed are smoothed to better represent the trend that is visible in the raw data displayed in **Figure 5**.

The three way interactions are visualized in **Figure 6**. For display purposes only, in this figure the time curves of dilation for the filler items were subtracted from the curves of the two conditions (target-matching or target-mismatching cues). This was done to accentuate the effect of lexical competition, which was minimized in the fillers. Also for display purposes only, the curves are smoothed by means of a locally weighted regression function with a span of 0.5. Increased pupil dilation as a function of mismatching cues in NS is illustrated in the steeper curve for target-mismatching items (upper panel). The far smaller effect of mismatching cues on pupil dilation in DS is visible in the smaller difference between the two curves displayed (bottom panel). The increase in pupil dilation as a function of lexical competition, as well as a function of mismatching cues is smaller than in NS.

##### Baseline 2

**FIGURE 7:**
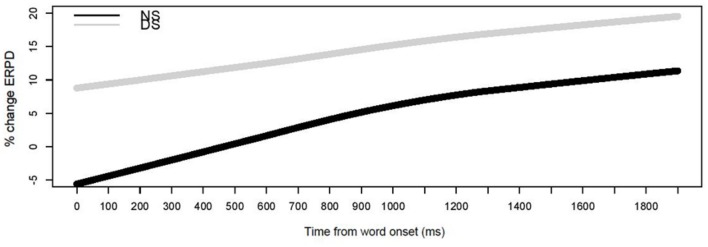
**Effect of listening to the sentences on mental effort, pupil dilation curves over time relative to baseline 2**.

**Figure 7** displays ERPD curves for DS and NS for baseline 2. For display purposes only, the curves are smoothed by means of a locally weighted regression function with a span of 0.5. The curves for both NS and DS differed from each other in all three terms. The first term of the curve function, describing the intercept [χ^2^(2) = 48.99, *p* < 0.001], the second term, describing the slope [χ^2^(2) = 85.24, *p* < 0.001], and the third term, describing the curvature [χ^2^(2) = 28.9, *p* < 0.001]. Especially the intercept term for the two curves is important. The intercept captures the pupil size change due to participating in the experiment, regardless of whether the source of changes in pupil size was mental effort or engagement required by the task. The negative intercept value for NS represents the decrease in pupil dilation in subsequent trials due to participation in the experiment. The positive intercept for DS instead represents an increase in pupil dilation in subsequent trials due to participation in the experiment.

The ERPD curves with baseline 2 captured the fact that in NS listeners’ pupil dilation increased gradually, after the presentation of the target, reaching a peak only after 900 ms after the onset of the word. In the DS condition, however, pupil dilation was already increased at the onset of the target word. While the overall dilation was greater in DS, this pupil dilation curve shows a very even course over the entire analysis window. This suggests that contrary to NS, where lexical disambiguation is at the source of increased pupil dilation, in DS participation in the experiment itself causes pupil dilation. Baseline 2 does not allow singling out individual processes at the source of pupil dilation, but we attribute the difference in ERPD calculated with baseline 2 to the demands that performing the experiment with DS posed on the participants. For NS **Figure 7** shows that the task was not increasing mental effort throughout the experiment, and lexical competition appears to be the main source of increased pupil dilation for NS. For DS **Figure 7** shows an increase due to performing the experiment, and explains why the analysis based on ERPD calculated with baseline 1 suggested a reduced mental effort for DS. The task itself, processing speech, has led to increased mental effort.

We investigated how signal degradation that simulates speech transmitted via CIs alters the time-course of speech perception and the mental effort drawn upon during this course. To sum up, we find a similar course of lexical disambiguation between degraded and natural signals, with a main difference in the timing of integration of durational cues, and the timing of resolution of lexical competition. Furthermore we find an increase in pupil dilation for listeners presented with NS, which is time-locked to lexical competition, and perception of target-mismatching cues. A different pattern of mental effort was found for DS, with pupil dilation not increasing as a function of lexical processing but due to the presentation with DS throughout the experiment. Increased effort in processing DS appears to have its sources at the pre-lexical level, while increased pupil dilation in NS has its source in lexical processing.

## Discussion

Our results from the conjunct analysis of gaze fixations with pupil dilation show different timing in the processing of DS at pre-lexical and lexical levels. At the pre-lexical level these timing differences seem to be the result of automatic versus more effortful processing of the signal. At the lexical level these timing differences appear to be the consequence of processing at the pre-lexical level with the corollary of different constrains on the selection of lexical candidates. For DS, increased mental effort has its source at the stages of pre-lexical processing, which further complicates the lexical processing. The finding of increased pupil dilation due to mismatching acoustic cues in NS, however, points to a possibly different recruitment of mental resources for natural versus degraded speech.

### Lexical Competition in Natural Speech

For natural stimuli, the gaze fixation results replicate the study by Salverda et al. (2003): durational differences between phonologically overlapping syllables in longer versus shorter words immediately modulate listeners’ lexical interpretations. Our recordings of pupil dilation show that mental resources are engaged during lexical access, and in particular when mismatching acoustic cues make listeners revise their initial lexical hypothesis. The small albeit significant increase in pupil size due to lexical competition in the cue-matching condition, together with the stronger increase in pupil size as a response to mismatching cues, shows how quickly speech perception can engage additional mental resources. With no effort perceived by the listener, this effect remains unnoticed in optimal listening conditions. Optimal conditions, i.e., conversation among native-speaker and normal-hearing (NH) listener in acoustically favorable surroundings, are rarely warranted in day-to-day communications. Although speech perception is commonly described as an automatic process, it likely draws on additional mental resources more often than not.

Increased processing or elevated activation of brain regions (Zekveld et al., 2014) correlates with pupil dilation, but it is not possible to make a clear-cut distinction between the processes that contribute to pupil dilation. We cannot strictly tell apart the sources of the observed pupil dilation for NS. Increased pupil dilation with mismatching cues could reflect mental effort. Increased dilation due to lexical competition in the cue matching condition can, however, also reflect the involvement of attentional processes rather than effort. There is evidence for attentional resources taking part in the automatic processing of speech (Wild et al., 2012; Wöstmann et al., 2015). In Posner’s (1992) framework, there is also the notion of the perceptual and cognitive system to be supported by autonomous attentional shifts that are automatically triggered by the stimulus as, in our case, the speech signal. Our results for NS support the interpretation that the incoming signal initiates speech perception automatically, but will draw on additional mental resources for the processing at pre-lexical or lexical levels, depending on the sort of degradations in the signal. According to the model of attention by Kahneman (1973), distribution of attentional resources is closely related to mental effort, with automatic tasks requiring less attentional resources. Processing of native speech becomes automatic through exposure, through the fine attunement of the perceptual system during speech development, and through extensive experience with the signal. The stimuli in our experiment misled our participants to a spurious lexical hypothesis by providing them with misleading cues. At first glance, this seems a rather artificial situation, but it is not completely unfamiliar, as it may occur for instance while communicating with foreign accented speech. Processing of foreign accented speech might affect the timing of pre-lexical and lexical processing in a similar way as our experimental condition. The less well-timed processing of speech may then recruit more mental resources.

The timing of speech processing appears to be crucial for the seamless automatic transfer of information from pre-lexical to lexical levels of analysis. The processing of early post-sensory but pre-lexical levels of speech perception is likely to be constrained by the capacity of the auditory sensory memory (Crowder, 1993) that limits the retention of acoustic details. Successful processing at this level facilitates quick lexical access, and swift resolution of lexical competition is necessary for the mapping of signal to meaning (Cleland et al., 2012). Well-timed transition of information between pre-lexical and lexical levels appears to conceal the engagement of attentional resources in speech perception. Our study thus stresses the need for a better understanding of the role of the timing window for the interaction between sensory, pre-lexical and lexical processing of speech.

In line with this interpretation are more recent findings on speech perception and attention. Winkler et al. (2005) report that pre-attentive processes of feature binding in auditory perception may require attentional processes for acoustically rich and complex stimuli when processed under time pressure. Similarly, Sörqvist et al. (2012) have shown that a non-auditory memory task diverts listeners’ attention to task-irrelevant speech sounds, which then also modulates their sensory processing, as captured in the Auditory Brainstem Response. Regarding the lexical processing of speech, Gaskell et al. (2008) showed in a series of experiments that early stages of speech processing can take place without drawing upon central resources, but accessing the meaning of words creates a bottleneck, which sets limit on the processing of subsequently presented words (Cleland et al., 2012). The magnitude of this limitation was modulated by the demands that a specific word poses on lexical competition, i.e., the similarity of the word to other words. As argued by Cleland et al. (2012) the access to the meaning of words occurs during a limited time window. In line with this, our results with NS did not show an increase in mental effort due to the task in the experiment (as captured in ERPD measured with baseline 2) since the speech materials were processed within the necessary time limitations. Still we observed increased pupil dilation due to mismatching cues in the signal, and we interpret this as a targeted engagement of automatic attentional resources rather than mental effort. A separation of sources that contribute to pupil dilation is however, still object to research.

### Lexical Competition in Degraded Signals

For DS, our results show that lexical competition was slower, prolonged and led to a less certain lexical decision. We also observed a reduced or delayed sensitivity to the durational cue, no increase in pupil dilation due to lexical competition or mismatching cues, and increased pupil dilation due to the demands of the experiment, which main task consisted of listening to speech. This last finding is in line with previous results (Zekveld et al., 2014; Winn et al., 2015) showing increased pupil dilation when listening to DS. We interpret our results as showing a bottom–up cascading effect of degradation. The small delay in uptake of the durational cues, which is visible also in the comparison between NS and DS in target-matching condition, carried over to high-level lexical stages of processing, accumulating additional delay during lexical competition. This accumulated uncertainty resulting from ill-timed processing at pre-lexical and lexical levels will have to be compensated for with increased demands on listeners’ working memory. Speech needs to be processed in real time, and delayed mapping of signal to meaning will not only make listeners entertain multiple interpretations of the spoken message, but will also limit their predictive processing of speech (Wagner et al., 2016).

The lack of sensitivity to durational cues can partly be explained by the nature of the degradation. The reduction of the spectrotemporal details from NS likely disrupts the binding of acoustic features into categories and reduces neuronal synchronization (Anderson et al., 2010) on the physiological level. Pre-attentive processes involved in the binding of acoustic features into auditory objects, such as phonemes or syllables, are also subjects to practice and experience, as is suggested by superior pre-attentive processing of musicians (Koelsch et al., 1999). Our participants were not experienced with the degradation prior to the experiment. Furthermore, attention to acoustic events appears to be also guided by spectrotemporal details that occur in NS (Ding et al., 2014; Wöstmann et al., 2015). Finally, the lack of naturally occurring consequences of coarticulation may smear out the acoustic features, which in natural signal are the binding elements of phonetic categories within words. All these factors contribute to the slower integration of acoustic features in the formation of perceptual objects such as syllables or words.

The slower progress of information between pre-lexical and lexical stages is also fortified by the fact that the signal does not resemble listeners’ mental representations. Lyxell et al. (1998) argue in a study with users of CIs that long-term deprivation of auditory sensory information before implantation may deteriorate the long-term representation of speech. In the present study it was the speech signal that was degraded, while the mental representation of our NH listeners was intact. A mismatch between the mental representations and the signal was present in our experiment nevertheless. Our results show that even short-term exposure to degraded signals affects its mapping to mental representations, by slowing it down. In addition, the less constrained mapping of signal to mental representations on the pre-lexical level has consequences for the processing on the lexical level.

Our results show that it is more difficult to revise built-up lexical expectations upon hearing DS signals. The delay on pre-lexical levels might have opened up the opportunity to build up stronger, and in this case, misleading lexical hypotheses about the word that was being processed. This explanation is supported firstly by the observed prolonged lexical competition, and secondly by the uncertainty about the lexical decision after disambiguating acoustic information was presented in DS. In line with this, Lash and Wingfield (2014) present evidence for an auditory analog to the Bruner–Potter effect. Bruner and Potter (1964) showed that recognition of an image presented in a progressive way from blurred to clear is slowed down relative to a singular presentation of a clear image. An unclear object leads participants to build up multiple hypotheses about the identity of an image, and rejecting several hypotheses requires longer than it takes for a single better-cued hypothesis to develop. In analogy to this, auditory presentation with degraded signal compromise its immediate processing and passing on to higher evaluation levels, causing listeners to hang on to spurious lexical hypotheses.

While we argue that the source of effort is the pre-lexical processing, there are also alternative explanations for the lack of an additive effect of lexical competition on pupil dilation for degraded signals. Firstly, it is likely that pupil dilation was not able to capture or differentiate additive effects of lexical competition and listening to DS. Secondly, the attentional resources that a listener can draw upon may be depleted by the attention directed toward the processing of degraded signals. A third explanation is that delayed reception of acoustic cues in degraded signals obscures lexical competition and alters the more targeted engagement of attentional resources found in NS. The processing effort found in natural signals would then not be comparable to the effort evoked by lexical competition for degraded signals. Though the three explanations are not mutually exclusive, we believe that the fixation data combined with the pupil dilation data provide some support for the last explanation. The gaze fixations show that lexical competition is delayed and prolonged for degraded signals, and we see increased pupil dilation due to listening to DS. Listeners’ engagement in lexical competition may be gated by attentional resources, and a constant effortful processing may disengage the automatic attentional processes that are supposed to be driven by the signal, making lexical competition a less automatic process.

To our knowledge this is the first study that combined measures of time–course of speech perception, in gaze fixations, with mental effort, in pupil dilation. Even though the sources underlying pupil dilation are manifold and difficult to strictly separate, and more research is on the way to investigate these sources, we believe that our study offers a contribution to this search. Speech perception can be an effortful task, in particular for CI users, but also in every-day non-optimal interactions. Our study shows involvement of mental resources for processes that are fundamental to speech perception, and how well-adjusted timing of information processing can conceal this involvement. We attribute experience with the task, i.e., speech perception, to be at the source of well-timed flow of information between stages of speech perception. An intriguing research question for the future is whether early exposure to degraded signals will lead to similar fine adjustment of speech processing, for instance in CI users who were implanted within the first year of their life. Related to this is also the fundamental question of the role that spectrotemporal details play in the process of well-timed speech processing, and regulation of attentional resources.

## Author Contributions

The author AW developed the concept of this study, acquired the data, analyzed and interpreted the results, and wrote the paper. AW gives the final approval of the version to be published, and agrees to be accountable for all aspects of the work. The author PT contributed to the data acquisition, and data analysis, and revised critically the final version of this paper for important intellectual content. PT gives the final approval of the version to be published, and agrees to be accountable for all aspects of the work. The author DB enabled the data acquisition, contributed to the interpretation of the results, and critically revised previous and the final version of this paper for important intellectual content. DB gives the final approval of the version to be published, and agrees to be accountable for all aspects of the work.

## Conflict of Interest Statement

The authors declare that the research was conducted in the absence of any commercial or financial relationships that could be construed as a potential conflict of interest.
