# Supplementary material for: The Timing and Effort of Lexical Access in Natural and Degraded Speech
Source: Front Psychol. 2016 Mar 30;7:398. doi: 10.3389/fpsyg.2016.00398 (PMC4811892; doi:10.3389/fpsyg.2016.00398)
Supplement: Supplementary file 1 [file Data_Sheet_1.DOCX]

Supplementary Material

List of stimuli

| Target | Competitor | Distractors | |
| --- | --- | --- | --- |
| bakker | bak | pin | lama |
| beitel | bij | vos | trecher |
| bliksem | blik | hark | vissekom |
| bokser | bok | peer | snijplank |
| cocktail | kok | tang | schommel |
| doperwt | dop | hand | koekepan |
| eikel | ei | bier | bureau |
| hamster | ham | kraan | wasmachine |
| hendel | hen | loep | paperclip |
| kijker | kei | vaas | molen |
| knipsel | knip | bas | vogelnest |
| lampenkap | lam | web | fornhuis |
| leiding | lei | hand | pompoen |
| mantel | man | boor | ladenkast |
| panty | pen | mand | radijs |
| regenton | ree | haai | schoorsteen |
| rooster | roos | been | vergiet |
| schilder | schil | tol | microscoop |
| slager | sla | hoed | piano |
| snorkel | snor | pijl | waaier |
| taxi | tak | berg | helikopter |
| tegel | thee | kaas | ananas |
| torso | tor | slee | fakkel |
| veter | fee | pan | traktor |
| zebra | zee | stoel | fopspeen |
| kussen | kus | bel | panda |
